# OCT and Autofluorescence Phenotypic Features in Autosomal Dominant *RHO*-Associated Retinitis Pigmentosa Variants

**DOI:** 10.3390/vision10020021

**Published:** 2026-04-10

**Authors:** Christina Karakosta, Saoud Al-Khuzaei, Penny Clouston, Morag Shanks, Susan M. Downes

**Affiliations:** 1 Oxford Eye Hospital, John Radcliffe Hospital, Oxford University Hospitals NHS Foundation Trust, Oxford OX3 9DU, UK; christine.karakosta@gmail.com (C.K.);; 2 Nuffield Laboratory of Ophthalmology, Nuffield Department of Clinical Neuroscience, University of Oxford, Level 6 John Radcliffe Hospital, Headley Way, Oxford OX3 9DU, UK; 3 Oxford Medical Genetics Laboratories, Oxford University Hospitals NHS Foundation Trust, Oxford OX3 7LE, UK

**Keywords:** rhodopsin, *RHO*, fundus autofluorescence, hyperautofluorescent ring, OCT, retinitis pigmentosa

## Abstract

Background/Objectives: To describe retinal imaging characteristics and the natural history of rhodopsin (*RHO*)-associated autosomal dominant retinitis pigmentosa (ADRP) by evaluating ellipsoid zone (EZ) width loss and measuring the degree of constriction of the area within and including the hyperautofluorescent ring. Methods: Eighteen patients with molecularly confirmed *RHO* variants were retrospectively evaluated. EZ width on spectral-domain optical coherence tomography (SD-OCT) and the area within and including the hyperfluorescent ring on fundus autofluorescence (FAF) were measured. The correlation between EZ width and hyperfluorescent ring area was assessed using a linear mixed-effects model. Results: Mean best corrected visual acuity (BCVA) (logMAR) was 0.21 at baseline and 0.29 at last visit over a mean follow-up of 5 years. Nine patients presented with sectoral RP, eight with typical RP, and one with unilateral RP. The mean EZ width constriction rate was −93.43 µm/year (SD = 130.58), and the area within and including the hyperautofluorescent ring decreased by −0.54 mm^2^/year (SD = 0.50). A strong positive association was observed between the EZ width and hyperfluorescent ring area at baseline (β = 151.7 ± 17.9, *p* < 0.001) and at the final visit (β = 185.7 ± 18.2, *p* < 0.001). Conclusions: In this study, patients with *RHO*-associated ADRP appeared to show a relatively slow rate of progression. Quantitative imaging markers, such as EZ width and the area within and including the hyperautofluorescent ring, may offer potentially reproducible measures of disease progression. These imaging biomarkers could be useful as outcome measures in future natural history studies and therapeutic trials, pending further validation.

## 1. Introduction

The terms inherited retinal degeneration, dystrophy or disease (IRD) have been variously used to describe a group of genetically determined retinal conditions, including retinitis pigmentosa (RP) and macular dystrophies [1]. Retinitis pigmentosa (RP) is the most common type of IRD, with a prevalence of 1:3000 to 1:5000 [2]. RP is a clinically and genetically heterogeneous condition marked by widespread, gradual dysfunction and degeneration primarily affecting rod photoreceptors, with subsequent involvement of cone photoreceptors and the retinal pigment epithelium (RPE) [3,4]. The typical clinical RP triad includes bone spicule-like intraretinal pigment migration, waxy optic disc pallor and attenuated arterioles [5].

Symptoms typically include night blindness and progressive loss of visual field [4]. The first identified gene causing RP was rhodopsin (*RHO*) (OMIM: 180380) [6]. More than 246 pathogenic variants in *RHO* have been reported to date [7,8]. The majority of *RHO* gene mutations (30–40%) cause autosomal dominant retinitis pigmentosa (ADRP), while only a few rare variants have been associated with autosomal recessive *RHO*-associated RP and dominantly inherited congenital stationary night blindness [9,10,11].

The *RHO* gene maps to the long arm of chromosome 3 at 3q22.1, comprises five coding exons, and encodes the rod photoreceptor-specific protein rhodopsin. Rhodopsin is a member of the class A G protein-coupled receptor (GPCR) superfamily characterized by seven transmembrane α-helical domains [9]. The rhodopsin polypeptide consists of 348 amino acids and is structurally organized into three principal domains: a cytoplasmic domain involved in G-protein interaction, a transmembrane domain facilitating phototransduction through *retinal* (vitamin A derivative) binding, and an intradiscal (intraluminal) domain implicated in protein folding and trafficking [9].

Rhodopsin is produced in the rod inner segment, and it is then transported to the outer segment [10]. When rhodopsin absorbs light, 11-cis-retinal converts to all-trans-retinal, changing the protein’s structure and activating the phototransduction cascade in the outer segment [10].

High-resolution structural imaging has become central to the diagnosis, characterization, staging and monitoring of IRDs. Two of the most widely used and well-validated structural biomarkers are the ellipsoid zone (EZ) width on spectral-domain optical coherence tomography (SD-OCT) and fundus autofluorescence (FAF) patterns; in particular, the presence and size of a hyperautofluorescent ring seen in many types of RP, or areas of decreased/absent autofluorescence in macular dystrophies [12,13]. The EZ width provides a direct, repeatable surrogate of photoreceptor integrity. Quantitative EZ width metrics have therefore been used extensively in natural history studies and proposed as structural endpoints for interventional trials because of their relative sensitivity to change and good inter-observer reproducibility [12,13].

Fundus autofluorescence complements SD-OCT by demonstrating lipofuscin distribution, indicating metabolic changes at the level of the RPE. [14]. FAF is particularly useful to delineate disease extent and to quantify progressive loss of viable retina. FAF-derived measures show good repeatability and correlate with structural SD-OCT measures and with functional measurements such as retinal sensitivity and extent of loss of visual field in multiple IRD genotypes [13,14].

Structural imaging using SD-OCT and FAF was used to in the *RPE65*-associated retinal dystrophy (Voretigene neparvovec-rzyl—Luxturna^®^) trial as part of the natural history characterization and post-treatment follow-up [15]. At that time, functional measures were the primary endpoints for regulatory approval. Since then, the value of these objective structural measurements has been increasingly recognized for monitoring disease progression and therapeutic response [16].

Autosomal dominant RP generally follows a more benign course than autosomal recessive or X-linked RP. While several studies have characterized disease progression in retinitis pigmentosa using EZ width and FAF imaging, these have largely focused on mixed-genotype RP cohorts. To date, there has been little quantitative analysis of structural progression in *RHO*-associated autosomal dominant RP. To the best of our knowledge, this is the first study to measure longitudinal change in both EZ width and the hyperautofluorescent ring area specifically in RP patients with *RHO* variants.

## 2. Materials and Methods

This study was conducted in accordance with the Declaration of Helsinki and was approved by the local research ethics committee (Ethics Approval Reference: 08/H0302/96). Informed consent was obtained from all study participants.

Patients with a pathogenic variant in *RHO* were retrospectively identified from the Oxford University Hospitals Medical Genetics Laboratory. The diagnosis of RP was confirmed based on both clinical features and genetic testing.

The medical records of all patients with a pathogenic variant in *RHO* who had been reviewed in an ophthalmic genetics clinic were evaluated retrospectively. Clinical data collected included baseline age, age of onset, sex, family history, symptoms, and best corrected visual acuity (BCVA). The BCVA was recorded on Snellen and LogMAR charts and all BCVAs were converted to logMAR for the purposes of statistical analysis. Multi-modal imaging included Optos pseudo-color widefield imaging (Optomap A10022; Optos Ltd., Dunfermline, Scotland, United Kingdom.), short wavelength FAF (Spectralis; Heidelberg Engineering, Heidelberg, Germany), and SD-OCT imaging (Spectralis, Heidelberg Engineering, Heidelberg, Germany).

Fundus autofluorescence and spectral domain SD-OCT scans were assessed at the first visit (baseline) and the last available follow-up. Measurements were performed by using proprietary Heidelberg software (Heidelberg Eye Explorer—HEYEX, version 2.6.9). All scans were acquired using the Heidelberg system with the built-in reference scan functionality; for each visit, the baseline reference image was used to ensure consistent alignment of the horizontal line scan. In addition, automated eye tracking (TruTrack) was enabled throughout to maintain scan reproducibility across time points.

**Figure 1:**
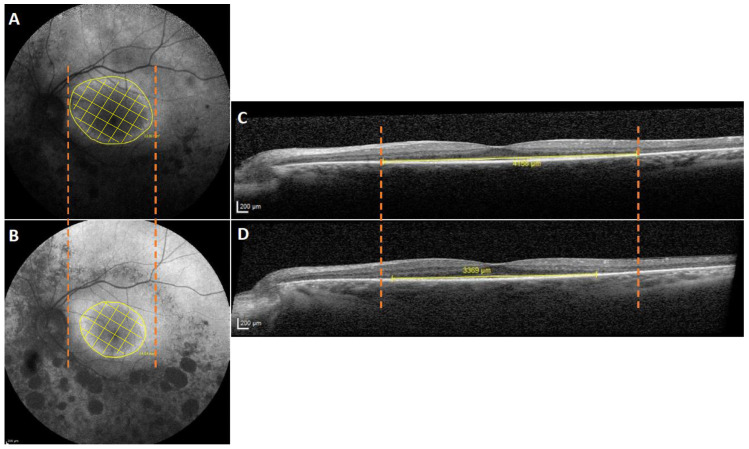
Representative imaging of RHO-associated retinitis pigmentosa. Fundus autofluorescence (FAF) image showing the area within and including the hyperautofluorescent ring at baseline (**A**) and at the last visit (**B**), highlighting progression (yellow crosshatch area within the orange dotted). The outer boundary of the ring, delineated in yellow, was used to measure the area within and including the hyperautofluorescent ring (mm^2^). Yellow crosshatching shows the area within the ring (**B**). Spectral-domain optical coherence tomography (SD-OCT) scan corresponding to the same eye, at baseline (**C**) and at the last visit (**D**). The yellow line indicates the measured ellipsoid zone width (μm), defined as the distance between the nasal and temporal edges of the EZ where it meets the retinal pigment epithelium (Scale bar: 200 μm). The orange dotted line delineates the location of the autofluorescent ring at baseline (panel (**A**)) compared with the last visit (panel (**B**)), to clarify the changes highlighting its centripetal contraction. These findings reflect inward migration of the autofluorescent ring, a recognized indicator of impending retinal pigment epithelium (RPE) degeneration, secondary to progressive loss of the intervening retinal tissue. A similar orange dotted line denotes the baseline width of the ellipsoid zone (panel (**C**)) relative to the last visit (panel (**D**)).

The hyperautofluorescent ring was defined by its externally delineable boundary on FAF imaging, corresponding to the visible transition between increased autofluorescence and the surrounding retina. Measurements were obtained using the built-in area measurement tool in the Heidelberg Eye Explorer (HEYEX), with manual delineation of the ring to calculate the enclosed surface area. Patients with an incomplete hyperautofluorescent ring, as seen in case 9 with sectoral RP, were excluded from the hyperfluorescent ring area analysis. On the SD-OCT, the EZ width was measured and defined as the distance between the nasal and temporal edges of the EZ where the middle of the EZ band merges with the retinal pigment epithelium (RPE) (Figure 1). This retinal distance was represented in degrees, assuming a conversion of 300 μm/° (Figure 1).

The EZ width and the area of hyperautofluorescent ring were independently measured by two researchers (CK, SA-K), and the average value was obtained from those two measured values and was used for further data analysis. Inter-observer agreement for all quantitative imaging measurements was assessed using the intraclass correlation coefficient (ICC) based on a two-way random-effects model for absolute agreement.

All analyses were carried out using the R programming language and the RStudio (version 2024.12.0+467) integrated development environment (IDE). Basic demographic characteristics of the patients were summarized with means and standard deviations (SDs) for normally distributed continuous variables or medians and interquartile ranges (IQRs) for skewed data. Normality of the variables was checked with the use of histograms, statistical moments (skewness and kurtosis) and the Shapiro–Wilk test. The hyperautofluorescent ring area was defined as the area within and including the hyperautofluorescent ring. To account for the inclusion of both eyes from the same patient and the resulting intra-subject correlation, linear mixed-effects models were used, with the patient included as a random effect. The association between hyperautofluorescent ring area and ΕΖ width was assessed at baseline and at the final visit using separate mixed-effects models. Statistical significance was defined as *p* < 0.05. A linear mixed-effects model was also used to assess the change in hyperfluorescent ring area and EZ width, accounting for the correlation between eyes of the same patient and adjusting for follow-up duration. To explore the functional relevance of structural changes, a linear mixed-effects model was used to assess whether changes in the hyperfluorescent ring area and EZ width were associated with changes in visual acuity. The model included change in the hyperfluorescent ring area and EZ width as fixed effects and a random intercept for patient ID to account for correlation between eyes.

Genetic testing was performed using next generation sequencing (NGS). Variant pathogenicity was investigated using in silico analysis (SIFT, PolyPhen-2, mutation taster, SpliceAI). The evolutionary conservation of novel missense variants was also investigated.

Genetic testing was performed at the Oxford Regional Genetics Laboratory, Oxford University Hospitals NHS Foundation Trust, as part of the routine patient care. Sequencing was performed using the method available at the Oxford Medical Genetics Laboratory at the time of patient presentation. Enrichment of the *RHO* gene was achieved using a customized HaloPlex enrichment system kit (Agilent Technologies, Santa Clara, CA, USA), designed to capture the coding exons and 10 bp of flanking introns of 111 retinal genes on the phenotype-led gene panel [17]. Next-generation sequencing (NGS) was performed using an Illumina MiSeq instrument (San Diego, CA, USA) with a MiSeq v3 kit, following the manufacturer’s instructions. Detected variants were confirmed by Sanger sequencing. Chromosome positions were based on the GRCh37/hg19 build, and nucleotide and protein numbering was based on transcript NM_000539, corresponding to the protein NP_000530.1 (*RHO*)**.** Variants were described according to Human Genome Variation Society (HGVS) nomenclature at both the cDNA and protein levels.

The potential pathogenicity of identified *RHO* variants was evaluated using four in silico analysis programs: MutationTaster [18], PolyPhen-2 (HumVar model) [19], SIFT [20] and REVEL. The combined annotation-dependent depletion (CADD) score was also calculated [21,22]. Missense variants were further assessed using Alphamisse [23], and predicted effects on pre-mRNA splicing were evaluated using SpliceAI [24,25]. Population allele frequencies were obtained from the Exome Aggregation Consortium (ExAC) and gnomAD databases [26], and variants with a minor allele frequency > 0.1% were excluded. Variants that were absent or extremely rare in population databases were considered supportive of pathogenicity.

All variants were classified according to the American College of Medical Genetics and Genomics (ACMG) guidelines, incorporating computational evidence, population frequency data, and previously reported clinical associations. Variants were considered novel if absent from the LOVD [27] and ClinVar [28] databases. To assess evolutionary conservation, multiple sequence alignment of rhodopsin protein sequences from Human (*Homo sapiens*) NP_000530.1; Chimpanzee (*Pan troglodytes*) XP_063663190.1; Cow (*Bos taurus*) NP_001014890.1; Rat (*Rattus norvegicus*) NP_254276.1; Mouse (*Mus musculus*) NP_663358.1; Chicken (*Gallus gallus*) NP_001384426.1; Frog (*Xenopus tropicalis*) NP_001090803.1 was performed using Clustal Omega (version 1.2.4) [29]. Conservation of amino acid residues affected by novel variants was evaluated across species to support functional relevance.

## 3. Results

This retrospective study included 35 eyes from 18 patients with a clinical diagnosis of RHO-associated RP, comprising 11 females and seven males. Of the 18 patients, nine had sectoral RP with an incomplete hyperfluorescent ring; these eyes were excluded from the hyperfluorescent ring area analysis. Additionally, SD-OCT scans were unavailable at baseline for five patients, who were therefore excluded from the EZ width analysis. Overall, 13 eyes from seven patients were eligible for the hyperfluorescent ring area analysis, and 25 eyes from 13 patients were eligible for the EZ width analysis. Correlation analysis between the two variables was conducted for the seven patients who had a complete hyperfluorescent ring and available FAF and SD-OCT scans at two distinct visits. Patients’ demographic and genetic details are summarized in Table 1 and Table 2. Information for each patient is presented in Table 3.

Thirteen pathogenic and likely pathogenic variants were identified in the *RHO* gene, including missense variants, in-frame deletions, and a canonical splice-site variant. Variants known to cause ADRP included p. (Pro23His), p. (Arg135Trp), and p. (Arg135Leu). These variants demonstrated consistent deleterious predictions across multiple in silico analysis tools and were absent from or extremely rare in population databases. Additional previously described missense variants, including p.(Leu68Arg), p. (Pro171Arg), p. (Glu181Lys), p. (Cys185Arg), p. (Asp190Asn), and p. (Pro347Leu), showed variable concordance across prediction algorithms but were classified as pathogenic or likely pathogenic according to ACMG criteria based on combined computational evidence, conservation, and rarity. A canonical splice-donor variant, c.936+1G>T, demonstrated a high predicted impact on splicing (SpliceAI score 0.99) and a high CADD score, consistent with a pathogenic loss-of-function mechanism.

**Figure 2:**
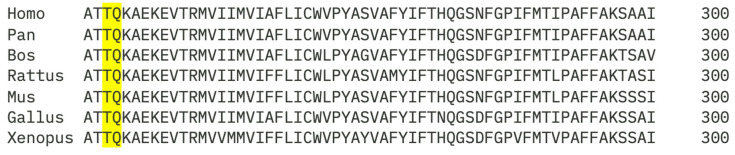
Sequence alignment of *RHO* across species for the novel c.728_730delCAC that resulted in the missense change in p.(Thr243Lys) and a deletion of Gln244 (highlighted in yellow) shows conservation of both these variants across different species.

We report a novel in-frame deletion, c.728_730delCAC, resulting in a combined protein-level effect of p. (Thr243Lys; Gln244del). This variant leads to the substitution of threonine at position 243 with lysine and the deletion of the adjacent glutamine residue at position 244. In silico tools predicted a deleterious effect on protein function, and the variant was absent from population databases. Both Thr243 and Gln244 were also shown to be conserved across all seven species. The affected residues are located within a conserved region of rhodopsin corresponding to the transmembrane domain, highlighting their functional importance. The strong evolutionary conservation of this region supports the pathogenic classification of the novel in-frame deletion (Figure 2).

**Figure 3:**
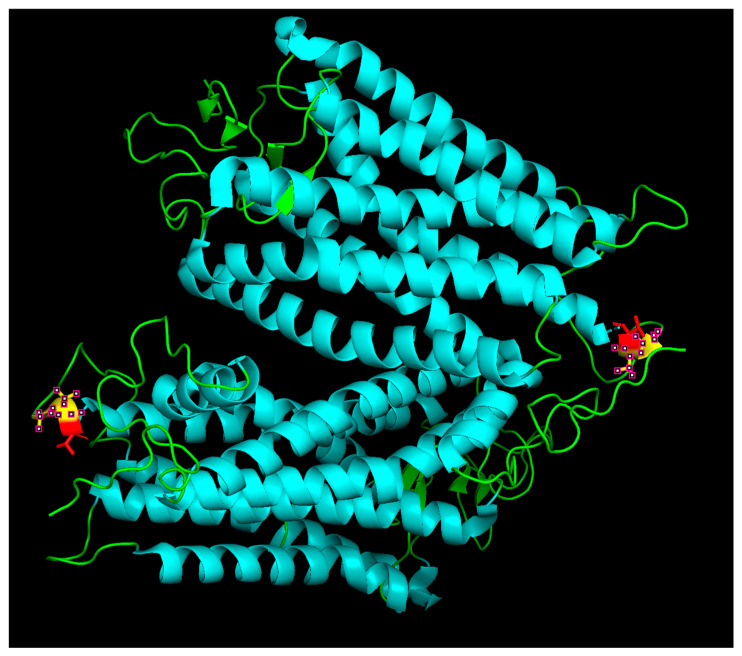
Structural visualization of the *RHO* Thr243Lys; Gln244del variant in PyMOL. The sixth transmembrane helix (TM6) of rhodopsin is shown in cyan cartoon representation. Residue Thr243, mutated to lysine, is highlighted in red, and neighboring residues are shown in yellow. The deletion of Gln244, shown as a gap due to software limitations, is expected to disrupt local helix packing. The combined effect of charge introduction and backbone perturbation supports a deleterious impact on rhodopsin stability.

Structural visualization of rhodopsin was performed using PyMOL (v2.5, Schrödinger, LLC, New York, NY, USA). The human rhodopsin crystal structure (PDB ID: 1U19) was used as a reference. The Thr243Lys substitution was modeled using the Mutagenesis Wizard, and Gln244 was deleted to approximate the in-frame deletion. Neighboring residues and the sixth transmembrane helix (TM6) were visualized to assess potential structural impacts of the variant (Figure 3). Structural modeling shows that Thr243 lies within TM6, and substitution with lysine introduces a charged residue into the hydrophobic helix. Deletion of Gln244 introduces a local gap, likely disrupting helix packing. These structural alterations support a deleterious effect on rhodopsin stability and folding.

Inter-rater reliability for the autofluorescent ring area measurements was ICC (2,1) = 0.999 (95% CI: 0.996–0.999), indicating very good agreement between reviewers. Inter-rater reliability for the EZ width measurements was ICC (2,1) = 0.9998 (95% CI: 0.9993–0.999), indicating very good agreement between reviewers.

**Figure 4:**
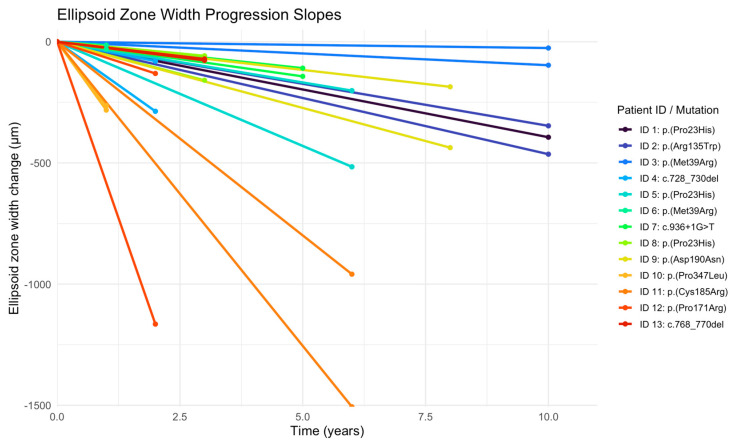
Plots of change in ellipsoid zone width over time for 25 eyes of 13 patients. In order to more clearly show the change over time, subjects have been centered to start at zero by subtracting baseline value from all of the values.

At baseline, the EZ width ranged from 945 µm to 6173 µm, with a mean of 3216 µm (SD = 1958). At the last follow-up, the EZ width ranged from 859 µm to 5987 µm, with a mean of 2895 µm (SD = 1903). At baseline, the area within and including the hyperautofluorescent ring ranged from 2.53 mm^2^ to 33.09 mm^2^, with a mean of 13.75 mm^2^ (SD = 9.77). At the last follow up, the area within and including the hyperautofluorescent ring ranged from 2.42 mm^2^ to 28.88 mm^2^, with a mean of 11.09 mm^2^ (SD = 8.60). The mean progression rate for the EZ width constriction was −93.43 µm/year (SD = 130.58 µm/year) (Figure 4).

**Figure 5:**
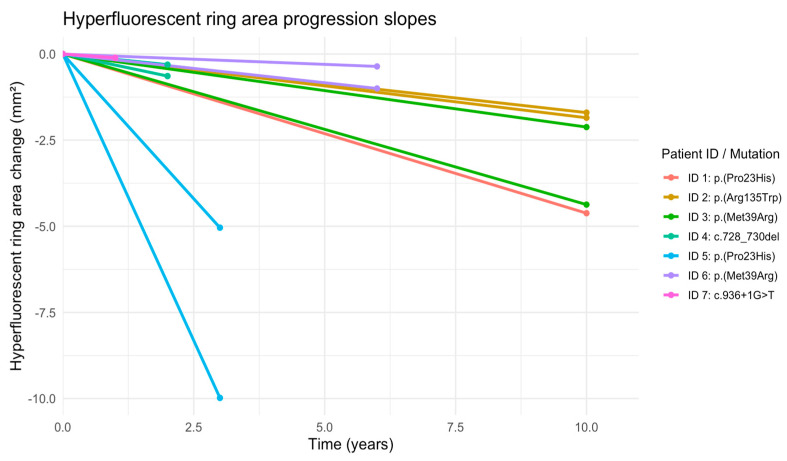
Plots of change in hyperfluorescent ring area over time for 13 eyes from 7 patients. In order to more clearly show the change over time, subjects have been centered to start at zero by subtracting the baseline value from all of the values. IDs 1–7 correspond to the same patients as in Figure 4.

The mean progression rate for the hyperfluorescent ring area constriction was −0.54 mm^2^/year (SD = 0.50 mm^2^/year) (Figure 5).

A strong positive association was observed between the EZ width and hyperfluorescent ring area at baseline, using a linear mixed-effects model to account for inter-eye correlation (β = 151.7 ± 17.9, *p* < 0.001). A similarly strong association was observed at the final visit between hyperfluorescent ring area and EZ width (β = 185.7 ± 18.2, *p* < 0.001).

For the hyperfluorescent ring area, the mean change from baseline to last follow-up was not significantly associated with follow-up interval (95% CI: −0.70 to 0.43 mm^2^). For EZ width, the change over time was also not significantly associated with follow-up interval (95% CI: −228.92 to 359.66 µm).

The change in the hyperfluorescent ring area (95% CI: −0.029 to 0.046) was not significantly associated with changes in visual acuity (*p* = 0.603). In contrast, there was a significant positive correlation between EZ width change and visual acuity change (r = 0.36, 95% CI: 0.08–0.59, *p* = 0.014), indicating that larger changes in EZ width were associated with greater changes in visual acuity. All variables of this cohort are summarized in Table 4.

**Figure 6:**
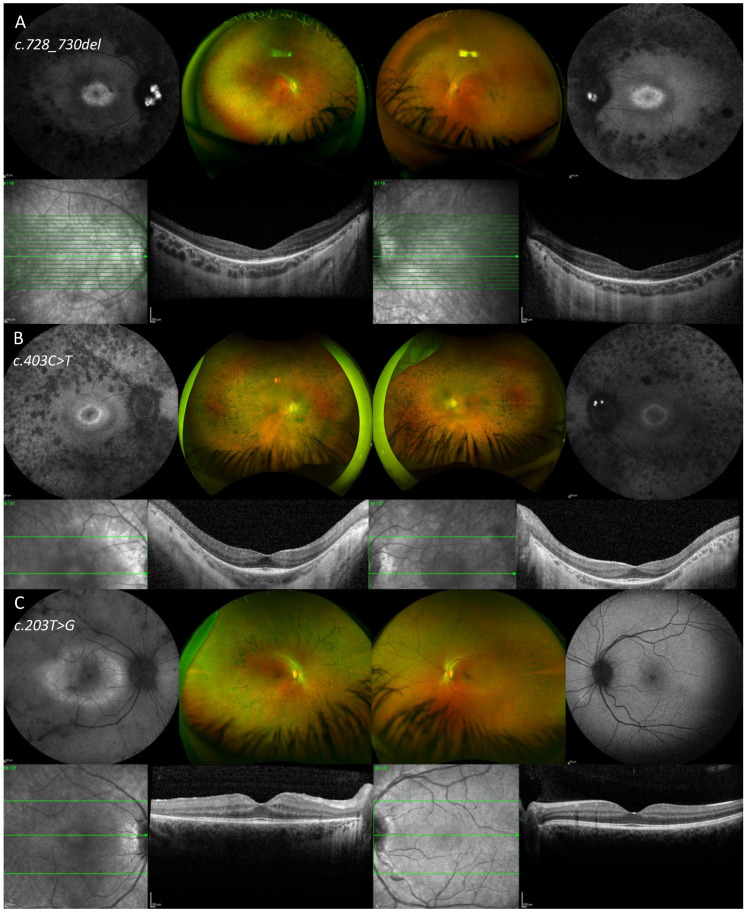
Fundus autofluorescence (FAF), color fundus images and optical coherence tomography of the right and left eye of two patients with typical retinitis pigmentosa (**A**,**B**) and one patient with unilateral retinitis pigmentosa (**C**). (**A**) In patient ID = 14, FAF imaging showed bilateral central raised macular autofluorescence (AF) and retinal atrophy within the posterior pole and pseudo-color fundus imaging showed retinal atrophy in the mid to far periphery with sparse bone spicule pigmentary deposition and vascular attenuation. Spectral Domain—Optical coherence tomography (SD-OCT) imaging showed preserved ellipsoid zone within the foveal region. (**B**) In patient ID = 7, FAF imaging showed bilateral rings of raised AF surrounding the foveal regions and retinal atrophy within the posterior pole. Pseudo-color imaging showed retinal atrophy in the mid periphery with bone spicule pigmentation. The SD-OCT image showed bilateral preserved outer retinal layers in the foveal area. (**C**) In the right eye of patient ID = 6, the FAF imaging showed a thick band/ring of raised AF in the macula, the pseudo-color imaging showed bone spicule pigmentary changes in the mid-periphery and the SD-OCT image showed preserved outer retinal layers in the foveal area. The left eye was within normal limits.

**Figure 7:**
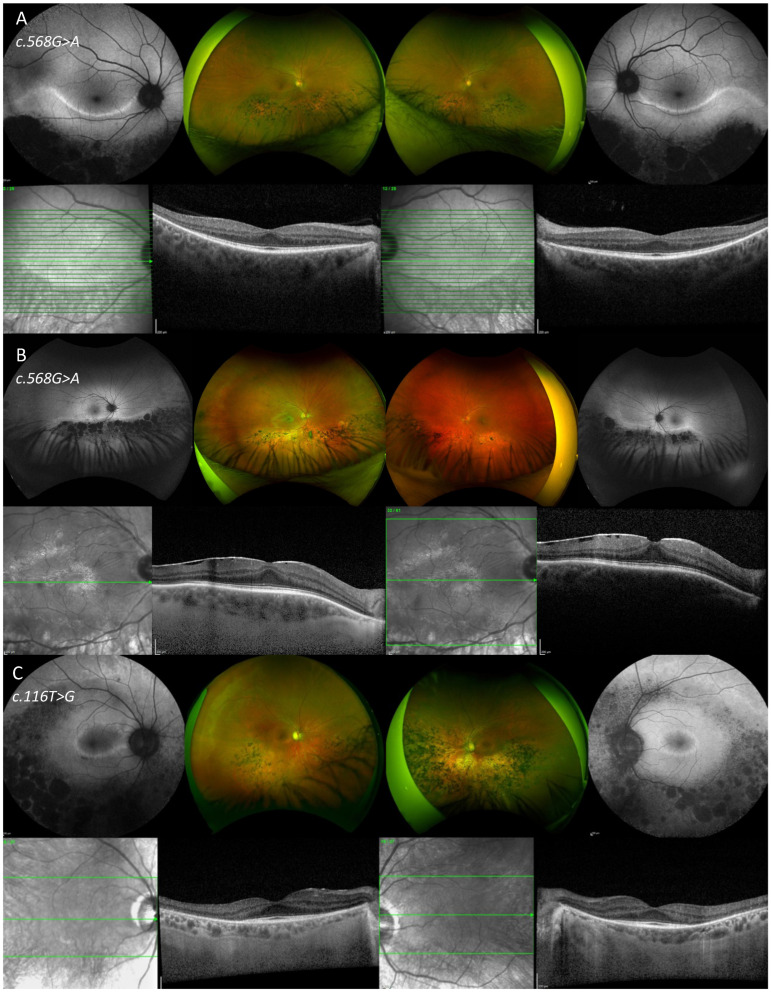
Fundus autofluorescence, color fundus images and optical coherence tomography of the right and left eye of three patients (ID = 13, 12 and 3, respectively) with sectoral retinitis pigmentosa (**A**–**C**).

In Figure 6 and Figure 7, color fundus imaging, FAF and SD-OCT are presented from six patients from the cohort.

## 4. Discussion

In this study, RHO-related ADRP progression was quantified using structural imaging biomarkers, including EZ width on SD-OCT B-scan and the area within and including the hyperautofluorescent ring on FAF. Both parameters showed a progressive decrease in size over time, with a mean reduction of 0.54 mm^2^/year for the hyperautofluorescent ring area and 93.43 µm/year for EZ width. These findings indicate measurable but relatively slow structural progression within this cohort.

To our knowledge, this is the first study to longitudinally assess both EZ width and hyperautofluorescent ring area, specifically in a genetically defined *RHO*-associated ADRP cohort. Previous studies have reported structural progression rates in more heterogeneous RP populations, often including mixed inheritance patterns and multiple genotypes. In those studies, faster rates of decline have generally been observed compared with those reported here, but direct comparisons should be interpreted with caution given differences in cohort composition, sample size, imaging methodologies, and follow-up duration. [30,31,32]. This difference likely reflects the underlying genetic mechanisms: in ADRP, many pathogenic variants, including those in *RHO*, exert dominant-negative or gain-of-function effects that lead to partial dysfunction of rod photoreceptors rather than complete loss [30,31,32]. It needs to be clarified that, while ADRP has been described in the literature as having a relatively mild clinical course compared with other inheritance patterns, the present study was not designed to directly compare progression rates across genetic subtypes. Therefore, the relatively slow progression observed in this cohort should not be attributed solely to the inheritance pattern or underlying molecular mechanisms, and further comparative studies would be required to clarify these relationships.

By contrast, autosomal recessive disorders often result from biallelic loss-of-function variants causing complete absence of functional protein, leading to earlier onset, faster photoreceptor degeneration, and more severe visual impairment [30,31]. Other studies assessing disease progression in mixed or recessive RP cohorts have reported faster structural decline rates compared with our *RHO*-related ADRP cohort [32,33,34,35]. Takahashi et al. assessed the RP disease progression measured as a function of the progressive loss of EZ width on OCT B-scan, constriction of the area within and including the hyperautofluorescent ring on FAF, and ring thickness on the enface OCT over time [36]. The study included nine patients with ADRP (2 *KLHL7*-, 4 *RHO*-, 2 *RP1*-, 1 *PRPF8*-related cases), 11 ARRP (6 *USH2a-*, 3 *PDE6A-*, 1 *CRB1-*, 1 *MAK1*-related case), 1 XLRP (RDGR-related case) and three syndromic RP cases with Usher syndrome (1 *USH2a*-, 1 *MYO7A*- and 1 *CLRN1*-related case). In this study, they reported that the width of the hyperfluorescent ring decreased at a mean change of 0.5 (SD 0.4) mm^2^ per year, the area within and including the hyperautofluorescent ring constricted with a mean change of 0.9 (0.98) mm^2^ per year mm^2^ and the EZ width constricted by 123 μm (SD 63) per year [36]. Sujirakul et al. described an EZ width decreasing rate of 140 μm (5.2%) per year [37]. A previous study reported that the EZ width decreased at an average rate of 130 μm (4.9%) per year [37]. Our study showed a significantly slower rate of progression compared to the previously reported AD RP studies, which can be explained by the heterogeneous nature of their cohorts (variants in different genes reported). By contrast, our study focused only on *RHO*-related ADRP, which typically exhibits a later onset and milder progression than autosomal recessive or X-linked RP (Table 5).

The quantitative imaging markers evaluated in this study—EZ width and surface of area within and including the hyperautofluorescent ring—demonstrated consistent longitudinal change, supporting their potential utility as structural indicators of disease progression in RHO-associated ADRP. However, given the retrospective design, limited sample size, and variability in follow-up duration, these findings should be considered preliminary.

Although structural changes in hyperfluorescent ring area and EZ width were observed over time, only EZ width changes were significantly associated with changes in visual acuity in our mixed-effects analysis. The lack of association of the hyperfluorescent ring area change is likely attributable to the small number of eyes included in this analysis.

In slowly progressive conditions, detecting meaningful change over short time intervals may be challenging. Further validation in larger, prospective cohorts with standardized imaging protocols is needed to determine the reproducibility and sensitivity of quantitative imaging markers across different disease stages and whether structural progression reliably predicts functional decline.

*RHO*-associated AD RP is known to exhibit phenotypic variability, including differences in age of onset, rate of progression, and the extent of macular preservation among affected individuals [40]. The variable disease course in *RHO*-ADRP is also influenced by variant-specific molecular mechanisms. Pathogenic *RHO* variants have been categorized into several functional classes based on their biochemical and cellular effects, including protein misfolding and endoplasmic reticulum (ER) retention (Class II), mis-trafficking to the outer segment (Class I), constitutive activation of rhodopsin (Class III), and altered post-translational stability or degradation (Class IV–V) [41,42]. Variants that cause severe misfolding and ER stress tend to produce earlier-onset, more rapidly progressive disease, whereas those that retain partial protein function or induce milder trafficking defects are associated with slower degeneration and longer preservation of central vision [43]. Although prior studies have proposed genotype–phenotype correlations based on these variant-specific molecular mechanisms, such relationships were not evaluated in the present study and remain an important area for future investigation. Integrating molecular classification with quantitative imaging may help refine prognostic assessment, but this approach requires further study.

Understanding these molecular subtypes is increasingly important in the context of therapeutic development, as emerging strategies such as allele-specific suppression, gene editing, and pharmacological chaperones may have differing efficacy depending on mutation class. Future genotype–phenotype correlation studies that integrate molecular classification with quantitative imaging endpoints will be critical to refine prognostic assessment and optimize patient selection for targeted therapies.

The current study should be interpreted within the context of its limitations. The modest sample size and retrospective design may influence estimates of progression rate, especially for analysis of the area within and including the hyperautofluorescent ring, which included a subset of patients (13 eyes from seven patients). In addition, variability in follow-up duration and inter-device variability could affect quantitative imaging measurements. Future multi-center prospective studies with standardized imaging protocols would allow more robust modeling of progression dynamics in *RHO*-associated RP.

## 5. Conclusions

In conclusion, the progression of structural retinal changes in *RHO*-associated autosomal dominant retinitis pigmentosa was quantified by measuring both the area within and including the hyperautofluorescent ring as well as the EZ width over time. The area within and including the hyperautofluorescent ring decreased at a mean rate of 0.54 mm^2^/year, while the EZ width decreased at a mean rate of 93.43 µm/year. A strong correlation was observed between EZ width and hyperautofluorescent ring area at both baseline and the last follow-up, while only EZ width changes were significantly associated with changes in visual acuity. Future work, including a direct comparison of progression rates in different causative genes, would further characterize the relative prognosis of *RHO*-ADRP and inform both clinical counseling and the design of genotype-specific therapeutic trials.

## Figures and Tables

**Table 1:** Demographic data of the cohort.

Variable	Mean Value
LogMAR VA (baseline)	0.21 (SD = 0.25)
LogMAR VA Last follow up	0.29 (SD = 0.34)
Age at baseline (years)	39.2 (SD = 16.7)
Average follow up interval (years)	5 [Range: 1–10]

LogAMR = logarithm of the Minimum Angle of Resolution; VA = Visual Acuity; SD = Standard Deviation.

**Table 2:** Genetic details of the patients.

Variant	Protein Coding	Variant Effect	Mutation Taster	SIFT	PolyPhen-2	SpliceAI Score	Alpha Missense	REVEL	CADD	GnomAD	ACMG	Novel
c.68C>A	p.(Pro23His)	Missense	Deleterious	Affect function	Probably Damaging	0.00	Moderate pathogenic	Strong pathogenic	28.4	Not reported	Pathogenic	N
c.116T>G	p.(Met39Arg)	Missense	Deleterious	Tolerated	Benign	0.00	Supporting pathogenic	Supporting benign	21.8	Not reported	Pathogenic	N
c.203T>G	p.(Leu68Arg)	Missense	Deleterious	Affect function	Possibly damaging	0.00	Pathogenic	Supporting pathogenic	28.4	0.00006363	Likely pathogenic	N
c.403C>T	p.(Arg135Trp)	Missense	Deleterious	Affect function	Probably Damaging	0.00	Strong Pathogenic	Moderate pathogenic	27.5	Not reported	Pathogenic	N
c.404G>T	p.(Arg135Leu)	Missense	Deleterious	Affect function	Probably damaging	0.02	Strong Pathogenic	Strong Pathogenic	32	Not reported	Pathogenic	N
c.512C>G	p.(Pro171Arg)	Missense	Deleterious	Affect function	Probably damaging	0.00	Pathogenic	Supporting pathogenic	25.5	Not reported	Likely pathogenic	N
c.541G>A	p.(Glu181Lys)	Missense	Deleterious	Affect function	Benign	0.01	Pathogenic	Moderate pathogenic	29.4	Not reported	Pathogenic	N
c.553T>C	p.(Cys185Arg)	Missense	Deleterious	Affect function	Possibly damaging	0.02	Pathogenic	Moderate pathogenic	26	Not reported	Likely pathogenic	N
c.568G>A	p.(Asp190Asn)	Missense	Deleterious	Tolerated	Possibly damaging	0.02	Moderate pathogenic	Intermediate	25.5	0.000003977	Pathogenic	N
c.728_730delCAC	p.(Thr243Lys) p.(Gln244del)	AA deletion	Deleterious	Affect function	Probably damaging	0.02	Pathogenic	-	-	Not reported	Pathogenic	Y
c.768_770delCAT	p.(Ile256del)	AA deletion	Deleterious	-	-	0.07	-	-	-	Not reported	pathogenic	N
c.936+1G>T ^1^		Splice site	-	-	-	0.99	-	-	34	0.00001415	pathogenic	N
c.1040C>T	p.(Pro347Leu)	Missense	Deleterious	Affect function	Probably damaging	0.01	Intermediate	Intermediate	32	0.000003538	Pathogenic	N

ACMG Classification: American College of Medical Genetics and Genomics Classification; Y: Yes; N: No. ^1^ This variant is usually seen in autosomal recessive RHO-RP. No second variant was identified in this case, but the phenotype is consistent with typical RP.

**Table 3:** Patients’ IDs linked to variants.

ID	Variant	Phenotype	Sex	Age at Baseline
1	c.68C>A p.(Pro23His)	sectoral RP	M	65
2	c.116T>G p.(Met39Arg)	sectoral RP	M	63
3	c.116T>G p.(Met39Arg)	sectoral RP	M	43
4	c.116T>G p.(Met39Arg)	typical RP	F	56
5	c.116T>G p.(Met39Arg)	sectoral RP	F	37
6	c.203T>G p.(Leu68Arg)	Unilateral RP	F	14
7	c.403C>T p.(Arg135Trp)	typical RP	M	24
8	c.404G-T p.(Arg135Leu)	typical RP	M	38
9	c.512C>G p.(Pro171Arg)	sectoral RP	F	40
10	c.541G>A p.(Glu181Lys)	sectoral RP	F	15
11	c.553T-C p.(Cys185Arg)	sectoral RP	F	65
12	c.568G>A p.(Asp190Asn)	sectoral RP	F	18
13	c.568G>A p.(Asp190Asn)	sectoral RP	M	53
14	c.728_730del	typical RP	F	29
15	c.768_770del	typical RP	F	49
16	c.936+1G>T	typical RP	F	50
17	c.1040C>T p.(Pro347Leu)	typical RP	F	22
18	c.1040C>T p.(Pro347leu)	typical RP	F	26

**Table 4:** Summary of variables.

Variable	Min	Mean	Max
Hyperfluorescent ring area mm^2^ (Baseline)	2.53	13.75	33.09
Hyperfluorescent ring area mm^2^ (Last Visit)	2.42	11.09	28.88
Ellipsoid zone width (Baseline)	945	3216	6173
Ellipsoid zone width (Last Visit)	859	2845	5987
Hyperfluorescent ring area constriction rate (mm^2^/year)		−0.54	
Ellipsoid zone width progression rate (μm/year)		−93.43	

**Table 5:** Structural progression rates in retinitis pigmentosa genotypes across different studies.

Study	Gene(s)	Inheritance	EZ Width Decline Rate (µm/Year)	HF Ring Area Decline
Sujirakul et al. (2015) [37] N = 24	*PDE6A*, *PRPF31*, *USH2A*, *CNGB1*, *RHO*, *CRX*, *MYO7A*, *RPE65*, *CRB1*, *RGR*, *VLGR1*, *SAG*, *PROM1*, *RPGR*, *RP1*, *MYH11*, *MYOM1*, *PDE6B*	AR, AD, XL	130	147 μm (0.51°)/year (horizontal diameter)
Cabral et al. (2017) [34] N = 81	*USH2A*, *PDE6B*, *PDE6A*, *CNGB1*, *MERTK*, *MAK*, *NPHP1*, *EYS*, *CRB1*, *RGR*, *RHO*, *PRPF31*, *RP1*, *USH2A*, *GPR98*, *PCDH15*, *RPGR*	AR, AD, XL	140	NA
Jauregui et al. (2019) [32] N = 96	*USH2A*, *PDE6β*, *EYS*, *PDE6α*, *CDHR1*, *CNGB1*, *DHDDS*, *KIZ*, *MAK*, *MERTK*, *MYO7A*, *C21ORF2*, *CERKL*, *FAM161A*, *GPR98*, *IFT140*, *NPHP1*, *REEP6*, *SPATA7*, *TULP1*, *RHO*, *RP1*, *PRPF31*, *KLHL7*, *IMPDH1*, *GUCA1B*, *NRL*, *PRPF8*, *PRPH2*, *RPGR*	AR, AD, XL	123	0.5 mm^2^/year
Tee et al. (2019) [38] N = 38	*RPGR*	XL	233	0.67 mm^2^/year
Takahashi et al. (2019) [36] N = 24	*KLHL7*, *PRPF8*, *RP1*, *RHO*, *USH2A*, *PDE6A*, *PDE6B*, *MAK1*, *CRB1*, *RPGR*, *CLRN1*, *MYO7A*	AR, AD, XL	123	0.5 mm^2^/year
Heyang et al. (2025) [39] N = 55	*USH2A*	AR	0.063	NA
Present study N = 13/N = 7	*RHO*	AD	93.43	0.54 mm^2^/year

Ellipsoid zone (EZ) width and hyperautofluorescent (HF) ring area decline rates are shown for different RP genotypes. AD = Autosomal dominant; AR = Autosomal recessive; XL = X-linked; N = number of patients.

## Data Availability

All data generated or analyzed during this study are included in this article. Further inquiries can be directed to the corresponding author.
